# *Lonicera japonica* Thunb. Ethanol Extract Exerts a Protective Effect on Normal Human Gastric Epithelial Cells by Modulating the Activity of Tumor-Necrosis-Factor-α-Induced Inflammatory Cyclooxygenase 2/Prostaglandin E2 and Matrix Metalloproteinase 9

**DOI:** 10.3390/cimb46070433

**Published:** 2024-07-09

**Authors:** Hsi-Lung Hsieh, Ming-Chin Yu, Yu-Chia Chang, Yi-Hsuan Wu, Kuo-Hsiung Huang, Ming-Ming Tsai

**Affiliations:** 1 Graduate Institute of Health Industry Technology, Research Center for Chinese Herbal Medicine, College of Human Ecology, Chang Gung University of Science and Technology, Taoyuan 333, Taiwan; hlhsieh@mail.cgust.edu.tw (H.-L.H.); ycchang03@mail.cgust.edu.tw (Y.-C.C.); yhwu03@mail.cgust.edu.tw (Y.-H.W.); 2 Department of Neurology, Chang Gung Memorial Hospital, Taoyuan 333, Taiwan; 3 Department of Chemical Engineering, R&D Center of Biochemical Engineering Technology, Ming Chi University of Technology, New Taipei City 301, Taiwan; 4 Department of General Surgery, New Taipei Municipal TuCheng Hospital, New Taipei 236, Taiwan; a75159@cgmh.org.tw; 5 College of Medicine, Chang Gung University, Taoyuan 333, Taiwan; 6 Department of General Surgery, Chang Gung Memorial Hospital, Taoyuan 333, Taiwan; 7 Department of Nursing, Division of Basic Medical Sciences, Chang-Gung University of Science and Technology, Taoyuan 333, Taiwan; khs586@cgmh.org.tw; 8 Department of Laboratory Medicine, Section of Clinical Serology and Immunology, Chang Gung Memorial Hospital, Taoyuan 333, Taiwan

**Keywords:** *Lonicera japonica* Thunb., matrix metallopeptidase-9, tumor necrosis factor-*α*, cyclooxygenase-2

## Abstract

Gastric inflammation-related disorders are commonly observed digestive system illnesses characterized by the activation of proinflammatory cytokines, particularly tumor necrosis factor-α (TNF-α). This results in the induction of cyclooxygenase-2 (COX-2)/prostaglandin E_2_ (PEG_2_) and matrix metallopeptidase-9 (MMP-9). These factors contribute to the pathogenesis of gastric inflammation disorders. We examined the preventive effects of *Lonicera japonica* Thunb. ethanol extract (Lj-EtOH) on gastric inflammation induced by TNF-α in normal human gastric mucosa epithelial cells (GES-1). The GES-1 cell line was used to establish a model that simulated the overexpression of COX-2/PGE_2_ and MMP-9 proteins induced by TNF-α to examine the anti-inflammatory properties of Lj extracts. The results indicated that Lj-EtOH exhibits significant inhibitory effects on COX-2/PEG_2_ and MMP-9 activity, attenuates cell migration, and provides protection against TNF-α-induced gastric inflammation. The protective effects of Lj-EtOH are associated with the modulation of COX-2/PEG_2_ and MMP-9 through the activation of TNFR–ERK 1/2 signaling pathways as well as the involvement of c-Fos and nuclear factor kappa B (NF-κB) signaling pathways. Based on our findings, Lj-EtOH exhibits a preventive effect on human gastric epithelial cells. Consequently, it may represent a novel treatment for the management of gastric inflammation.

## 1. Introduction

*Lonicera japonica* Thunb. (Lj) is a Chinese herbal plant (homology of medicine and food) of which the flower, seeds, and leaves have been used for medicine since ancient times. Many studies have been conducted to evaluate the activity of Lj extract, and its various effects on human health have been established, including antioxidative, antibacterial, anti-inflammatory, antiviral, anticancer, and various other activities [1]. The dried flower buds of Lj may be prepared as tea which is known for its detoxifying and soothing effects [2].

Recent studies have provided evidence suggesting a progressive rise in gastric inflammation-related disorders, which may be attributed to a multitude of factors, such as an aging population, the prevalence of chronic pain, and the increased use of nonsteroidal anti-inflammatory drugs (NSAIDs) or diverse chemotherapeutic medications. These factors contribute to a rapid escalation of gastric inflammation-related disorders, which can worsen gradually over time [3,4].

Gastric inflammation-related disorders arise from the chronic erosion of gastric mucosal epithelial cells, which surpasses the regenerative capacity of these cells. This imbalance leads to the manifestation of gastric inflammation, fibrosis at the site of injury, and thinning of the gastric wall and may result in complications, such as gastric bleeding, gastric perforation, or the development of gastric cancer (GC) [5].

The etiology of gastric inflammation includes impaired or recurrent inflammation of the gastric mucosal epithelial tissue, which culminates in the activation of cytokines and chemokines. This subsequently results in the activation and infiltration of macrophages, neutrophils, monocytes, and other immune cells into the gastric tissue. The immune cells produce proinflammatory cytokines, such as tumor necrosis factor alpha (TNF-α), interleukins such as IL-1, IL-6, IL-8, and IL-10, and cell adhesion proteins like circular dichroism 44 (CD44) and intercellular adhesion molecule-1 (ICAM-1). The aforementioned factors serve as triggers for the production of inflammatory proteins, including cyclooxygenase-2 (COX-2) and matrix metalloproteinase-9 (MMP-9), within the mucosal lining in GC. The pathophysiological features of gastric inflammation arise from stimulation by immune cells, such as neutrophils, monocytes, and macrophages, and their infiltration into the gastric tissue. This leads to the development of inflammatory responses in the gastric mucosal epithelial cells, subsequent damage to the gastric mucosa, and the degradation of gastric secretory glands, among others [6,7,8].

TNF-α regulates diverse biological functions, including fever, inflammation, immune response, apoptosis induction, lipid metabolism, tumorigenesis, and viral replication [9,10]. Several studies indicate that increased expression of TNF-α or TNF-α polymorphisms has been associated with an augmented susceptibility to chronic atrophic gastritis, metastasis [11], tumorigenesis [12], and GC in patients [13,14]. Harris et al. [15] and Fan et al. [16] reported similar findings, supporting the concept that the pathogenesis of *H. pylori*-associated gastroduodenal diseases involves the upregulation of inflammatory TNF-α production.

COX-2 is an enzyme that is involved in the biosynthesis of prostaglandin (PG)-endoperoxide. Potter et al. [17] observed marked upregulation of COX-2 in neoplastic sites and GC with inflammation. Moreover, *Helicobacter pylori* infection stimulates Toll-like receptor (TLR)/MyD88 and COX-2/PGE_2_ pathways, resulting in NF-κB activation with different inflammatory responses in tumor tissues [18]. We demonstrated that *H. zeylanica*-E2 inhibits TNF-α-induced activation of the inflammatory cytosolic phospholipase A2 (cPLA2)/COX-2/PGE_2_ response in GC cells [19]. Therefore, the COX-2/PGE_2_ system likely serves as a central pathological mediator of gastric inflammation-related disorders.

MMP-9 contributes to the development of various human malignancies by facilitating wound healing, cell migration, angiogenesis, and tumor progression through collagen IV degradation in the basement membrane and extracellular matrix [20]. The upregulation of MMP-9, mediated by the nuclear factor kappa B (NF-κB) signaling, may promote gastric inflammation and actively contribute to the progression of GC [21]. Moreover, the findings in our previous study indicated that under normal conditions, quercetin exerts protective effects in GES-1 cells by inhibiting TNF-α-induced MMP-9 upregulation [22].

In gastric mucosal epithelium cells, Lj has been shown to exhibit gastroprotective properties following TNF-α-induced damage. However, the detailed underlying mechanisms are still unknown. Here, we elucidated the potential preventive properties of Lj on gastric mucosal epithelial cells in response to TNF-α-induced damage, with a specific focus on determining its effects on COX-2/PGE_2_ and MMP-9 signaling pathways. We used GES-1 cells under normal conditions to assess the molecular processes and immune-inflammatory reactions regulated by TNF-α.

## 2. Materials and Methods

### 2.1. Preparation of Lonicera japonica Thunb. Extracts

The dry herb of Lj was provided by Sheng Chang Pharmaceutical Co., Ltd. (Taipei, Taiwan), where the Lj powder was extracted using a Dionex™ ASE™ 350 extractor (Thermo Fisher Scientific Inc., Foster City, CA, USA). Lj powder (5 g) was placed into 66 mL container equipped with a stainless-steel frit and a cellulose filter at the bottom. Extraction was carried out using H_2_O or 95% EtOH as the solvent at a temperature of 100 °C (H_2_O extract, Lj-H_2_O) or 50 °C (EtOH extract, Lj-EtOH), a static extraction time of 15 min, 1 extraction cycle, and a constant pressure of 1500 psi. The extracts were collected in glass vials and evaporated on a rotary evaporator. Finally, the Lj extracts were stored at −20 °C until use.

### 2.2. Cell Line and Cell Culture

GES-1 cells were obtained from Xiamen University (China). The cells were incubated in RPMI-1640 medium (Invitrogen, Carlsbad, CA, USA) supplemented with 10% fetal bovine serum (Invitrogen, Carlsbad, CA, USA), 100 IU/mL penicillin G (Sigma–Aldrich, Jefferson City, MO, USA), 100 mg/mL streptomycin sulfate (Sigma–Aldrich, Jefferson City, MO, USA), and nonessential amino acids (Sigma–Aldrich, Jefferson City, MO, USA). Cells were cultured at a temperature of 37 °C in a humidified incubator containing 5% CO_2_ as previously described [19]. The cells were treated with Lj-H_2_O (1 μg/mL) and Lj-EtOH (1 μg/mL) extracts as well as inhibitors of TNF-α (TNF-α antagonist, No. SC-356159) (1 μM), ERK1/2 (U0126, No. SC-222395) (1 μM), c-Fos (Tanshinone IIA (TSIIA) (10 μM), No. SC-200932), NF-κB (BAY 11-7082, No. SC-202490) (10 μM), COX-2 (NS-398, No. SC-58635) (5 μM) (Santa Cruz Biotechnology, Dallas, TX, USA), or MMP9 (MMP9i) (No. 15942-500) (5 μM) (Biomol, Kelayres, PA, USA). The compounds were administered to the cultured cells 1 h before the addition of TNF-α (30 ng/mL) (AFL210) (R&D Systems, NE Minneapolis, MN, USA).

### 2.3. Matrix Metallopeptidase (MMP) Zymography

Cells were treated with Lj extracts (1 μg/mL) and inhibitors targeting TNF-α antagonist (1 μM), NF-κB (BAY 11-7082) (10 μM), ERK1/2 (U0126) (1 μM), c-Fos (TSIIA) (10 μM), COX-2 (NS-398) (5 μM), or MMP9 (MMP9i) (5 μM) to the cultured cells 1 h before the addition of TNF-α (30 ng/mL). The medium was collected and combined with 5 × nonreducing sample buffer. The resulting mixture was subjected to electrophoresis on a 10% gel containing 0.15% gelatin. Following the electrophoresis process, the gel was incubated, stained, and de-stained for the analysis of gelatinolytic activity by comparing horizontally aligned white bands against a blue backdrop [23].

### 2.4. Cell Counting Kit-8 (CCK-8) Assay

GES-1 cells were seeded into 96-well plates with a cell density of 5000 cells per well. The cells were incubated for 24 h in a humidified incubator containing 5% CO_2_. Lj extracts were diluted to prepare a range of concentrations (0, 0.1, 0.5, 1, 5, or 10 mg/mL), which were added to the cells and incubated for another 24 h. After adding 10 μL of CCK-8 kit reagent (MedChemExpress Ltd., Monmouth Junction, NJ, USA) to each well and incubating at 37 °C for 1 h, the cell density was measured using a microplate reader at 450 nm (SpectraMax i3; Kelowna International Scientific, NewTaipei, Taiwan) [24]. To evaluate cell viability, the ratio of viable cells to the control group was quantified.

### 2.5. Quantitative Reverse Transcription–PCR (qRT-PCR)

GES-1 cells were seeded into 9 cm dishes and incubated at 37 °C for 24 h. The cells were treated with TNF-α (30 ng/mL) for 0, 16, 20, or 24 h. Total RNA was isolated from the cells using TRIzol reagent (Invitrogen, Carlsbad, CA, USA), and the RNA concentration was determined using a Nano100 Micro-Spectrophotometer (CLUBIO; Taipei, Taiwan). cDNA synthesis was carried out using the iScript cDNA Synthesis Kit manufactured by Bio-Rad Laboratories, Inc (Hercules, CA, USA), and amplification was carried out using SsoFast EvaGreen Supermix and a thermal cycler (iCycler; Bio-Rad Laboratories, Foster, CA, USA). To measure the expression of *MMP9* and *COX2* mRNA in GES-1 cells, qRT-PCR was conducted by using *GAPDH* as the internal control [25] through the utilization of the CFX Connect Real-Time PCR system. The primer sequences were as follows: For *COX2*, the sense primer was 5′-CGGTGAAACTCTGGCTAGACAG-3′ and the antisense primer was 5′-GCAAACCGTAGATGCTCAGGGA-3′. For *MMP9*, the sense primer was 5′-AGTTTGGTGTCGCGGAGCAC-3′ and the antisense primer was 5′-TACATGAGCGCTTCCGGCAC-3′. For *GAPDH*, the sense primer was 5′-ACAGTCAGCCGCATCTTCTT-3′ and the antisense primer was 5′-GACAAGCTTCCCGTTCTCAG-3′. The quantification of gene expression levels was carried out by ΔΔCt methodology, whereby Ct denotes the average threshold cycle value.

### 2.6. Western Blot Analysis

The treated cells were subjected to either no intervention or exposure to Lj extracts (1 μg/mL) or targeted inhibitors of TNFα (1 μM), NF-κB (BAY 11-7082) (10 μM), ERK1/2 (U0126) (1 μM), c-Fos (TSIIA) (10 μM), COX-2 (NS-398) (5 μM), or MMP-9 (MMP9i) (5 μM) to the cultured cells 1 h before the addition of TNF-α (30 ng/mL). The cells were lysed using a lysis buffer at 4 °C as previously described [26]. Equivalent amounts of lysate were loaded onto a 10% SDS-PAGE gel, separated by electrophoresis, and transferred to polyvinylidene fluoride (PVDF) membranes (GE Healthcare Biosciences, Chicago, IL, USA). The specific primary antibodies were as follows: anti-phospho-ERK1/2 (#4377), anti-ERK2 (#9108), anti-phospho-c-Fos (#5348), anti-c-Fos (#2250), anti-phospho-NF-κB (p65) (#3033), anti-NF-κB (#8242), anti-COX-2 (#4842), or anti-glyceraldehyde-3-phosphate dehydrogenase (GAPDH) (#2118) (Cell Signaling Technology, Danvers, MA, USA). After washing, the membranes were incubated with anti-mouse (sc-7056) or anti-rabbit (sc-5054) antibodies conjugated with horseradish peroxidase (HRP) (Santa Cruz Biotechnology, St. Louis, MO, USA). The relative levels of ERK1/2, c-Fos, NF-κB, and COX-2 expression were normalized to the expression of GAPDH. Images of the bands were captured using a UVP BioSpectrum 500 Imager from UVP, Inc. (UVP LLC, Upland, CA, USA). Quantification of the results was carried out by densitometry analysis using UN-SCAN-IT gel computer software (Silk Scientific, Vineyard, UT, USA).

### 2.7. Cell Migration Assay

GES-1 cells were seeded in 6-well plates and cultured until confluence. The medium was replaced with a serum-free RPMI medium and incubated for 24 h. The cell monolayer was incised with disposable cell scrapers and any dislodged cells were removed with PBS. Following pretreatment with Lj extracts (1 μg/mL) or inhibitors targeting TNFα (1 μM), NF-κB (BAY 11-7082) (10 μM), c-Fos (TSIIA) (10 μM), ERK1/2 (U0126) (1 μM), MMP-9 (MMP9i) (5 μM), or COX-2 (NS-398) (5 μM) for 1 h, serum-free medium with or without TNF-α (30 ng/mL) was added to each well. Image acquisition was carried out by microscopy using a digital camera (Olympus, Tokyo, Japan) at two time points: 0 and 24 h. For each time point, a set of four-phase images was acquired and the average of these images was normalized to the initial image recorded at 0 h. The normalized values were subsequently averaged across all experimental conditions. The data represent three distinct and separate experimental trials [24].

### 2.8. Immunofluorescence Stain

GES-1 cells were seeded onto coverslips in culture plates. The cells were either untreated or treated with Lj extracts (1 μg/mL) and exposed to NF-κB (BAY 11-7082) (10 μM) or ERK1/2 (U0126) (1 μM) inhibitors to the cultured cells 1 h before the addition of TNF-α (30 ng/mL). After fixing and staining with an anti-NF-κB antibody, the coverslips were subsequently counterstained with 4′,6-diamidino-2-phenylindole (Vector Laboratories, Newark, CA, USA) as previously described [24]. The cells were imaged by a fluorescence microscope (Leica Microsystems, Wetzlar, Germany).

### 2.9. Luciferase Assay

The reporter plasmids pGL4.44 [*luc*2P/AP1 RE/Hygro] (accession number E411A) and pGL4.32 [*luc*2P/NF-κB-RE/Hygro] (accession number E849A) were acquired from Promega (Madison, WI, United States), which were used to express human AP-1 and NF-κB response elements in cells. These two plasmids were introduced into normal GES-1 cells separately with pGL4.73 [hRluc/SV40] (accession number E691A) plasmid using TurboFect Transfection Reagent (Thermo Fisher Scientific, Waltham, MA, USA) as previously described [22]. The cells were treated with Lj extracts (1 μM) along with inhibitors of TNF-α (TNF-α antagonist) (1 μM), ERK1/2 (U0126) (1 μM), or NF-κB (BAY 11-7082) (10 μM) added to the cultured cells 1 h before the addition of TNF-α (30 ng/mL). After treatment, the cells were collected, lysed, and centrifuged, and aliquots of the supernatant were evaluated for promoter activity using the dual-luciferase assay system. The activity of firefly luciferase was normalized to that of *Renilla* luciferase.

### 2.10. ELISA

The PGE_2_ protein level in the medium from cells untreated or treated with TNF-α or LjH_2_O (1 μg/mL), LjEtOH (1 μg/mL), TNF-α antagonist (1 μM), and COX2 inhibitor (5 μM) 1 h before the addition of TNF-α (30 ng/mL) at different time points was measured using a human protein PGE_2_ enzyme immunoassay kit (Enzo Life Sciences, Farmingdale, NY, USA). Fluorescence intensity was measured at 450 nm using a SpectraMax i3 microplate reader (Kelowna International Scientific Inc., New Taipei, Taiwan).

### 2.11. Statistical Analysis

The data represent a minimum of three separate trials and are presented as the mean ± standard error of the mean (SEM). Statistical analysis was carried out using a one-way ANOVA analysis by GraphPad Prism 6.0 computer software (GraphPad computer software, Inc., San Diego, CA, USA), supplemented by Tukey’s test to determine significant differences among multiple groups. *p* < 0.05 was considered statistically significant. The statistical methodologies were evaluated by Dr. Jian-Hao Chen, who is affiliated with the Estat Statistics Consulting Company (Taipei, Taiwan).

## 3. Results

### 3.1. COX-2 and MMP-9 Expression upon TNF-α Treatment in GES-1 Cells

**Figure 1:**
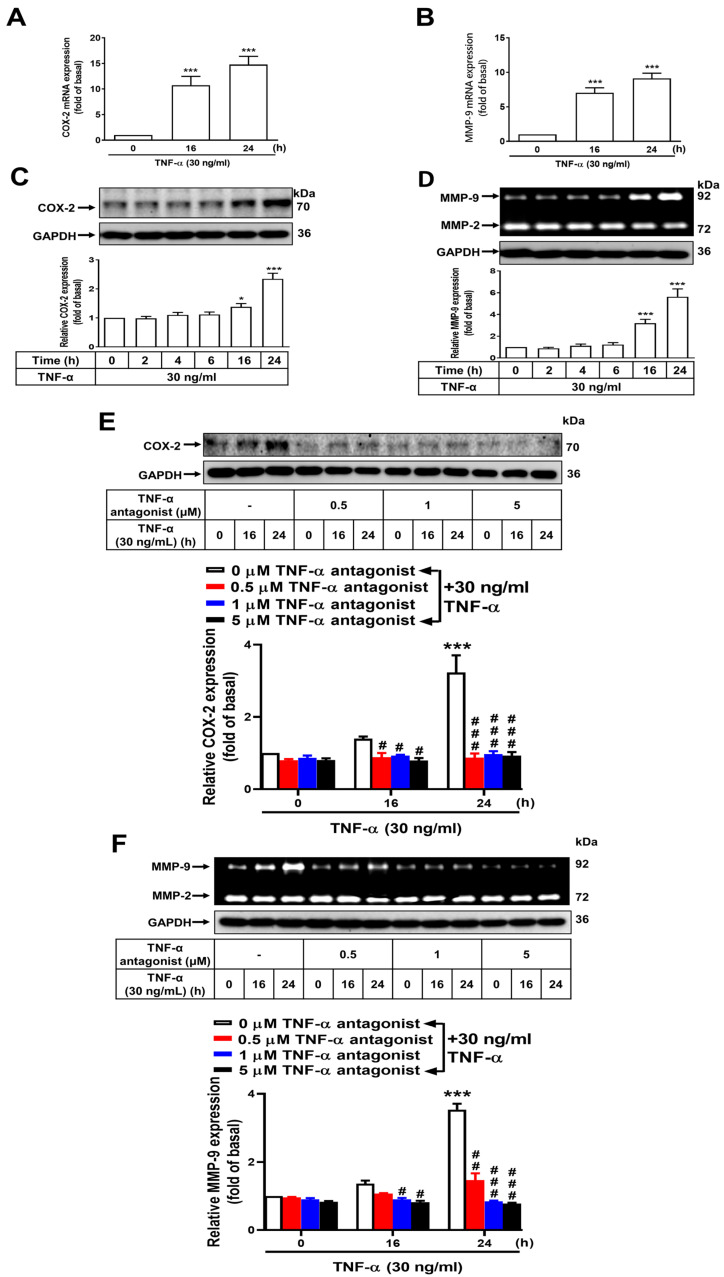
Induction of COX-2 and MMP-9 expression by TNF-α through the TNF-α receptor in normal GES-1 cells. Normal GES-1 cells were stimulated with TNF-α at a concentration of 30 ng/mL for 0, 16, or 24 h. The transcripts of (**A**) COX-2 and (**B**) MMP-9 were measured by qRT-PCR. In addition, (**C**) GES-1 cells were stimulated with TNF-α at a concentration of 30 ng/mL for 0, 2, 4, 6, 16, or 24 h and the expression of COX-2 was examined by Western blot analysis, (**D**) whereas the enzymatic activity of MMP-9 was assessed by gelatin zymography. The TNF-α antagonist effectively suppressed TNF-α-induced expression of (**E**) COX-2 and (**F**) MMP-9 in GES-1 cells. The data are presented as the mean ± SEM of three independent experiments. (* *p* < 0.05, *** *p* < 0.001 vs. control cells at 0 h; ^#^ *p* < 0.05, ^##^ *p* < 0.01, ^###^ *p* < 0.001 vs. TNF-α-stimulated cells).

The increased expression of MMP-9 through the NF-κB-dependent pathway has been implicated in the acceleration of gastric inflammation and the pathogenesis of GC, a condition characterized by poor survival [27,28,29]. Potter et al. reported that a marked activation of COX-2 occurs in inflamed neoplastic sites or GC [17]. By using qRT-PCR, COX-2, and MMP-9 mRNA were measured in normal GES-1 cells treated with TNF-α. After stimulation with TNF-α, a significant increase in COX-2 and MMP-9 mRNA levels was observed in a time-dependent manner at 16 and 24 h compared with the 0 h time point (*p* < 0.01 for all) (Figure 1A,B). To determine the relationship between TNF-α-activated COX-2 and MMP-9 in GES-1 cells, the cells were exposed to 30 ng/mL TNF-α for 0, 2, 4, 6, 16, or 24 h. Following treatment, COX-2 and MMP-9 protein levels were measured. As compared with the 0 h time point, TNF-α treatment (30 ng/mL) for 16 or 24 h resulted in the highest increase in COX-2 and MMP-9 expression (*p* < 0.05, *p* < 0.01 at 16 h, and *p* < 0.01, *p* < 0.01 at 24 h, respectively). The induction of COX-2 and MMP-9 by TNF-α occurred in a time-dependent manner (Figure 1C,D). The results indicate that COX-2 and MMP-9 expression in GES-1 cells are enhanced at both mRNA (transcriptional) and protein (translational) levels following TNF-α exposure. Next, we determined whether a TNF-α antagonist could suppress the expression of *COX2* and *MMP9* induced by TNF-α. The cells were further pretreated with a TNF-α antagonist at 0.5, 1, or 5 μM for 1 h followed by TNF-α exposure. There was a marked reduction in the expression of TNF-α-induced COX-2 in GES-1 cells in a time-dependent manner after 16 and 24 h of incubation compared with the control (*p* < 0.05 for all at 16 h and *p* < 0.01 for all at 24 h, respectively) (Figure 1E). Similarly, over 16 h and 24 h, MMP-9 expression was also decreased considerably in GES-1 cells upon TNF-α antagonist treatment compared with the 0 h measurement (*p* < 0.05 for 1 μM TNF-α antagonist, *p* < 0.05 for 5 μM at 16 h and *p* < 0.01, for all at 24 h, respectively) (Figure 1E,F). One hour of exposure before addition of the TNF-α antagonist at different concentrations is effective in the shortest time for gastric anti-inflammatory response (Figure 1E,F). In this study, pretreatment with drugs for one hour could provide a preventive effect.

### 3.2. Effect of Lonicera japonica Thunb. Ethanol Extract on the Expressions of TNF-α-Induced COX-2, PGE_2_, and MMP-9 in GES-1 Cells

**Figure 2:**
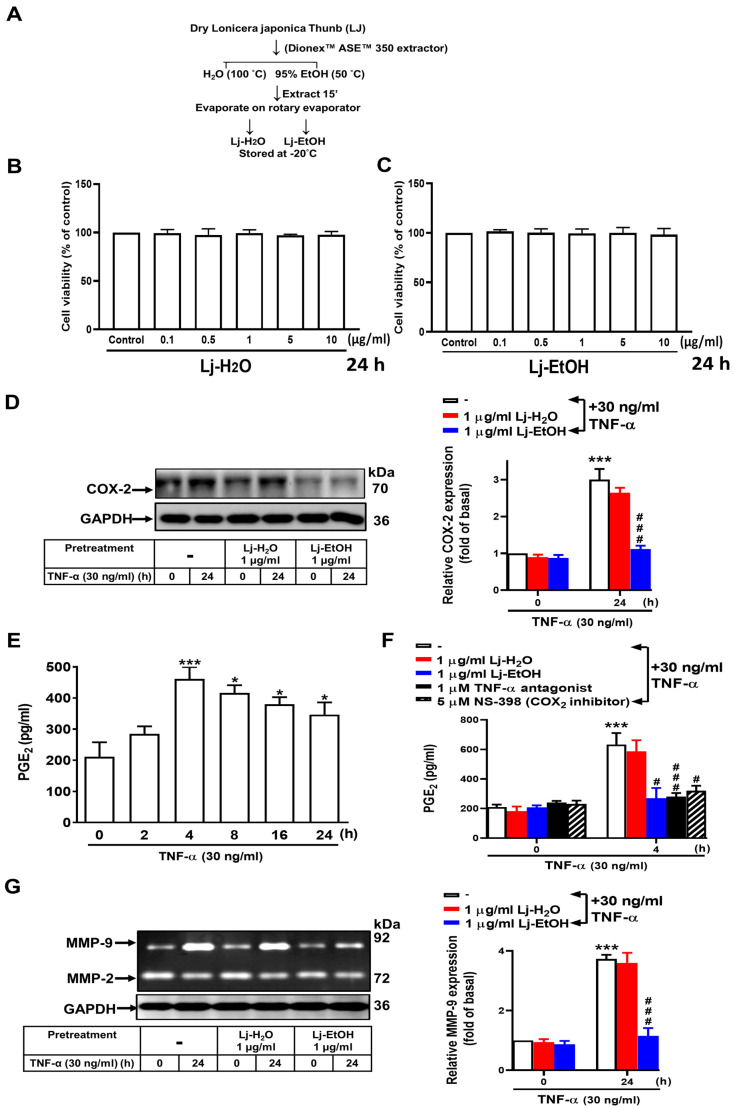
*Lonicera japonica* Thunb. ethanol extract exerts a suppressive effect on TNF-α-induced expression of PGE_2_, COX-2, and MMP-9 in normal GES-1 cells. (**A**) *Lonicera japonica* Thunb. extracts were prepared. (**B**) The effects of a water extract (Lj-H_2_O) and ethanol extract (Lj-EtOH) on the viability of normal GES-1 cells were evaluated. (**C**) GES-1 cells were treated with various concentrations of *Lonicera japonica* Thunb. extracts (0, 0.1, 0.5, 1, 5, or 10 μg/mL) for 24 h and cell viability was measured by CCK-8 assay. (**D**) The cells were divided into two groups: one for control and the other for treatment with Lj-H_2_O (1 μg/mL) or Lj-EtOH (1 μg/mL) for 1 h. TNF-α (30 ng/mL) was then added to both groups of cells. COX-2 was measured by Western blot analysis. (**E**) Quantitation of PGE_2_ protein was carried out using an enzyme-linked immunosorbent assay (ELISA) at 0, 2, 4, 8, 16, and 24 h of treatment. (**F**) Cells were treated with Lj-H_2_O (1 μg/mL), Lj-EtOH (1 μg/mL), TNF-α antagonist (1 μg/mL), or NS-398 (5 μM; COX-2 inhibitor). The conditioned medium was collected and PGE_2_ levels were measured by ELISA after 4 h. (**G**) The MMP-9 activity of GES-1 cells was measured by gelatin zymography after 24 h. The data are presented as the mean ± SEM of three independent experiments. (* *p* < 0.05, *** *p* < 0.001 vs. control cells at 0 h; ^#^ *p* < 0.05, ^###^ *p* < 0.001 as compared to TNF-α-treated cells).

Lj exhibits anti-inflammatory effects, as demonstrated in previous studies [30,31,32]. Water extracts (Lj-H_2_O) and ethanol extracts (Lj-EtOH) of *Lonicera japonica* Thunb. were prepared utilizing a Dionex™ ASE™ 350 Accelerated Solvent Extractor (Figure 2A). The viability of the extracts was evaluated in GES-1 cells by CCK-8 assay analysis. After adding the serial dilutions of Lj-H_2_O and Lj-EtOH extracts to the medium and incubating for 24 h in GES-1 cells, the viability of GES-1 cells was unaffected with Lj-H_2_O and Lj-EtOH at concentrations of up to 10 µg/mL (Figure 2B,C). To determine the effects on proinflammatory COX-2, PGE_2_, and MMP-9 expression caused by Lj extracts, GSE-1 cells were pretreated with 1 µg/mL Lj-H_2_O or Lj-EtOH. After treatment with 30 ng/mL TNF-α for 24 h in GES-1 cells, the levels of COX-2, PGE_2_, and MMP-9 proteins were assessed. In addition to COX-2 (Figure 1C), TNF-α (30 ng/mL) treatment for the indicated times also resulted in a marked upregulation of PGE_2_ protein compared with at 0 h (*p* < 0.01 at 4 h, *p* < 0.05 at 8 h, *p* < 0.05 at 16 h, and *p* < 0.05 at 24 h, respectively). Subsequently, expression of PGE_2_ was gradually reduced by the effects of TNF-α at 8 h and 16 h until 24 h. TNF-α treatment resulted in a significant increase in PGE_2_ levels over time (Figure 2E). Following this, we chose, at 4 h, this time point to continue the next inhibitor experiments (Figure 2F). Pretreatment with Lj-EtOH but not Lj-H_2_O resulted in a significant reduction in TNF-α-induced COX-2 protein (Figure 2D). Moreover, pretreatment with Lj- EtOH, TNF-α antagonist, and NS-398, but not Lj-H_2_O, in GES-1 cells resulted in a significant reduction in TNF-α-induced PGE_2_ protein compared with the control (*p* < 0.05 in Lj-EtOH, *p* < 0.01 in TNF-α antagonist, and *p* < 0.05 in NS-398 at 4 h, respectively), as shown in Figure 2F. The administration of Lj-EtOH resulted in a significant decrease in MMP-9 protein expression in GES-1 cells after 24 h (*p* < 0.01 for both) (Figure 2G). Comparing the two extracts, Lj-H_2_O and Lj-EtOH, only Lj-EtOH caused a decrease in the expression of proinflammatory proteins (COX-2, PGE_2_, and MMP-9) upon TNF-α stimulation in normal GES-1 cells. Lj-EtOH extracts exerted the most potent effect in gastric inflammatory response, and thus we chose Lj-EtOH extracts, not Lj-H_2_O extracts. These findings suggest that Lj-EtOH exhibits a unique and targeted mechanism of action.

### 3.3. The Role of ERK1/2 and c-Fos in TNF-α-Stimulated COX-2 and MMP-9 Expression in Normal GES-1 Cells

**Figure 3:**
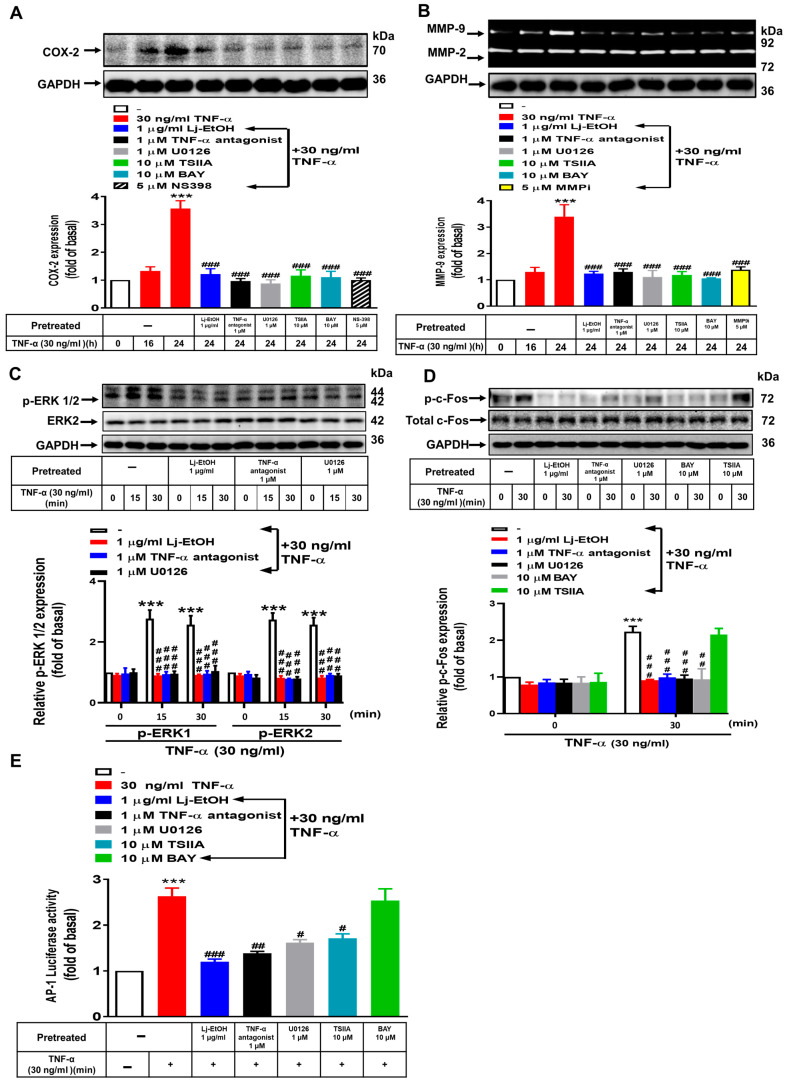
*Lonicera japonica* Thunb. ethanol extract exerts an inhibitory effect on TNF-α-induced COX-2 and MMP-9 expression in normal GES-1 cells by blocking the TNFR/ERK1/2/c-Fos pathway. (**A**,**B**) GES-1 cells were treated with TNF-α (30 ng/mL) for 24 h with a significant increase in COX-2 and MMP-9 expression. In drug pretreatment conditions, GES-1 cells were treated with Lj-EtOH (1 μg/mL), TNF-α antagonist (1 μM), U0126 (1 μM), TSIIA (10 μM), BAY 11-7082 (10 μM), and NS-398 (5 μM) or MMP9i (5 μM) for a duration of 1 h before the introduction of TNF-α; Subsequently, the cells were incubated with TNF-α for a period of 24 h. The level of COX-2 protein expression was determined by Western blot analysis (**A**), and the enzymatic activity of MMP-9 was determined by gelatin zymography (**B**). (**C**) GES-1 cells were treated with Lj-EtOH (1 μg/mL), TNF-α antagonist (1 μM), or U0126 (1 μM) for 1 h before the addition of TNF-α (30 ng/mL) for 0, 15, or 30 min. The phosphorylation of ERK1/2 (p-ERK 1/2) was measured by Western blot analysis. (**D**) GES-1 cells were treated with Lj-EtOH (1 μg/mL), TNF-α antagonist (1 μM), U0126 (1 μM), BAY 11-7082 (10 μM), or TSIIA (10 μM) for 1 h before the addition of TNF-α (30 ng/mL) for 0 or 30 min. The phosphorylation of c-Fos (p-c-Fos) was measured by Western blot analysis. (**E**) The GES-1 cell line was transfected with human AP-1–Luc response element reporter plasmids. The cells were then pretreated with Lj-EtOH (1 μg/mL), TNF-α antagonist (1 μM), U0126 (1 μM), TSIIA (10 μM), or BAY 11-7082 (10 μM) for 1 h. Following pretreatment, the cells were exposed to TNF-α for 1 h. Luciferase activity was measured to determine the activity of AP-1 and normalized to that of Renilla luciferase activity. The data are presented as the mean ± SEM of three independent experiments. (*** *p* < 0.001 vs. control cells at 0 h; ^#^ *p* < 0.05, ^##^ *p* < 0.01, ^###^ *p* < 0.001 as compared to TNF-α-treated cells).

Kim et al. [33] demonstrated that TNF-α-induced COX-2 and MMP-9 expression in human GES-1 cells is mediated through the mitogen-activated protein kinase (MAPK) pathway. We determined the underlying mechanism by which Lj-EtOH decreases the expression of COX-2 and MMP-9 upon TNF-α treatment in normal GES-1 cells. Treatment of GSE-1 cells with Lj-EtOH, TNF-α antagonist, U0126, TSIIA, BAY 11-7082, and NS-398 effectively inhibited TNF-α-induced COX-2 expression compared with the 24 h time point (*p* < 0.01 for all) (Figure 3A). Similarly, treatment with Lj-EtOH, TNF-α antagonist, U0126, TSIIA, BAY 11-7082, and MMP9i significantly inhibited the expression of TNF-α-stimulated MMP-9 protein in normal GES-1 cells compared with the 24 h interval (*p* < 0.01 for all) (Figure 3B).

**Figure 4:**
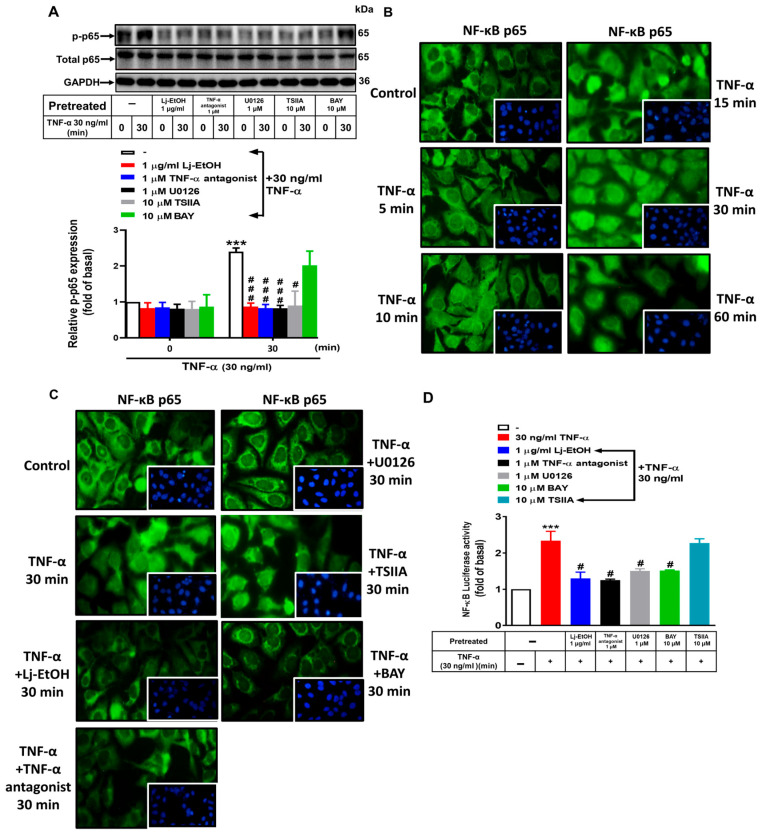
Activation of NF-κB (p65) upon TNF-α stimulation is inhibited by *Lonicera japonica* Thunb. ethanol extract in normal GES-1 cells (**A**) The cells were pretreated with 1 μg/mL Lj-EtOH, 1 μM TNF-α antagonist, 1 μM U0126, 10 μM TSIIA, or 10 μM BAY 11-7082 for 1 h followed by 30 ng/mL TNF-α for either 0 or 30 min. The phosphorylation of NF-κB was determined by Western blot analysis. (**B**) After treatment with 30 ng/mL TNF-α for 0, 5, 10, 15, 30, and 60 min, NF-κB phosphorylation in GES-1 cells was determined by immunofluorescence analysis. The green fluorescence is NF-κB (p65), and blue fluorescence are DAPI (nuclei). (**C**) The cells were treated with 1 μg/mL Lj-EtOH, 1 μM TNF-α antagonist, 1 μM U0126, 10 μM TSIIA, or 10 μM BAY 11-7082 for 1 h. Afterward, 30 ng/mL TNF-α was added for 30 min. The phosphorylation of NF-κB was determined by immunofluorescence analysis. The green fluorescence is NF-κB (p65), and blue fluorescence are DAPI (nuclei). (**D**) GES-1 cells were first transfected with human NF-κB response element reporter plasmids and then treated with 1 μg/mL Lj-EtOH, 1 μM TNF-α antagonist, 1 μM U0126, 10 μM TSIIA, or 10 μM BAY 11-7082 for 1 h. Afterward, 30 ng/mL TNF-α was added for 1 h, and luciferase activity was measured, using Renilla luciferase activity as normalization. The data are presented as the mean ± SEM of three independent experiments. (*** *p* < 0.001 vs. control cells at 0 h; ^#^ *p* < 0.05, ^###^ *p* < 0.001 compared to TNF-α-stimulated cells).

Additionally, phosphoproteins can be activated within the short term (several minutes); however, genes’ expression often takes several hours. p-ERK1/2, p-c-Fos, and p-p65 belong to the phospho-kinase proteins, which activate expression in the short term (mins), different from COX-2 and MMP9 proteins which show long-term expression (hours). Continuing our previous research [22], p-ERK1/2, p-c-Fos, and p-p65 showed a time course at 0, 5, 15, 30, 60 min by stimulation of TNF-α (24 h) [22]. Thus, p-ERK1/2 (Figure 3C), p-c-Fos (Figure 3D), and p-p65 (Figure 4) show the effect of TNF-α at 30 min (24 h). To further determine the role of ERK1/2 in the upregulation of COX-2 and MMP-9 expression following TNF-α induction, we used Lj-EtOH and two inhibitors, TNF-α antagonist and U0126. To gain insight into the effect of Lj-EtOH extracts, U0126, or TNF-α antagonist on ERK1/2 phosphorylation, the levels of ERK1/2 phosphorylation at different times (0, 15, or 30 min) were assessed by Western blot analysis. The phosphorylation of ERK1/2 induced by TNF-α was suppressed in a time-dependent manner following pretreatment with Lj-EtOH extracts, U0126, or a TNF-α antagonist (Figure 3C). The results indicated that the TNFR-ERK1/2 signaling pathway may facilitate the activation of COX-2 and MMP-9 expression upon TNF-α stimulation in normal GES-1 cells. Moreover, to determine the effect of Lj-EtOH, TNF-α antagonist, U0126, and BAY 11-7082 on c-Fos phosphorylation, the level of c-Fos phosphorylation following a 30 min TNF-α stimulation was assessed by Western blot analysis. All of the compounds exhibited significant inhibition on TNF-α-induced c-Fos phosphorylation in GES-1 cells compared with the control at 0 min, except TSIIA (Figure 3D).

The transcription factors AP-1 and NF-κB play an important role in modulating the promoter region of the human COX-2 and MMP-9 genes, in which they engage in interactions with cytokines and growth factors [34,35]. To determine the potential involvement of the above signaling pathways in decreased AP-1 promoter reporter activity caused by Lj-EtOH, a human AP-1 response element reporter assay was conducted. Treatment with Lj-EtOH led to a reduction in AP-1 reporter activity (Figure 3E). Taken together, the results indicate that Lj-EtOH decreases the expressions of TNF-α-induced MMP-9 and COX-2 in normal GES-1 cells through the TNFR-ERK1/2-c-Fos pathway.

### 3.4. The Effect of NF-κB on TNF-α-Stimulated COX-2 and MMP-9 Expression in Normal GES-1 Cells

Western blot analysis was carried out to determine whether Lj-EtOH, TNF-α antagonist, U0126, TSIIA, and BAY 11-7082 affected TNF-α-induced NF-κB (p65) phosphorylation. Thus, the phosphorylation of NF-κB (p65) was measured at various time intervals. Pretreatment with Lj-EtOH, TNF-α antagonist, U0126, TSIIA, and BAY 11-7082 resulted in the suppression of NF-κB phosphorylation (p-p65) upon TNF-α stimulation in a time-dependent manner (Figure 4A). An immunofluorescence stain was used to determine the effect of Lj-EtOH on the translocation of NF-κB from the cytoplasm to the nucleus. After exposure to 30 ng/mL TNF-α for 0, 5, 10, 15, 30, or 60 min, the gradual movement of NF-κB into the nucleus was observed, with the most pronounced effect occurring within 30 min. The translocation of NF-κB persisted for the entire 60 min assessment period (Figure 4B).

The pretreatment of TNF-α antagonist, U0126, BAY 11-7082, and Lj-EtOH reduced the TNF-α-stimulated translocation of NF-κB, excepting TSIIA (Figure 4C). A reporter plasmid assay containing a human NF-κB response element was further used to delineate the potential mechanism. The effect of Lj-EtOH treatment on the modulation of NF-κB promoter reporter activity was assessed in the presence of signaling inhibitors. The activity of the NF-κB promoter reporter was decreased following exposure to Lj-EtOH, TNF-α antagonist, U0126, and BAY 11-7082. There was no significant change in reporter activity in GES-1 cells treated with TSIIA upon TNF-α stimulation (Figure 4D). The results suggest that Lj-EtOH successfully diminishes the expression of COX-2 and MMP-9 induced by TNF-α in normal GES-1 cells through the TNFR/ERK1/2/c-Fos and NF-κB pathways.

### 3.5. The Antimetastatic Activity of Ethanol Extract from Lonicera japonica Thunb. Was Evaluated In Vitro

**Figure 5:**
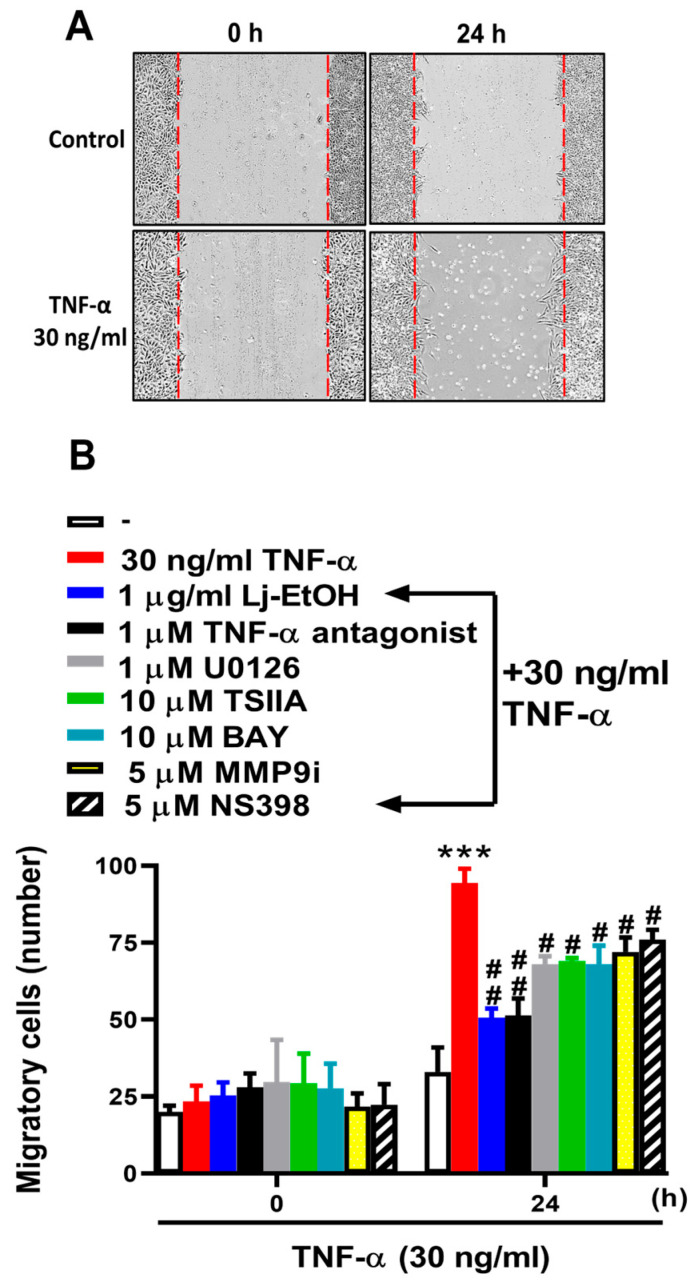
*Lonicera japonica* Thunb. ethanol extract exerts antimetastatic activities in vitro. (**A**) After reaching confluence and serum starvation for 24 h, GES-1 cells were pretreated with 1 μg/mL Lj-EtOH, 1 μM TNF-α antagonist, 1 μM U0126, 10 μM TSIIA, 10 μM BAY 11-7082 for 1 h, 5 μM MMP9i, or NS-398 for 1 h. To evaluate cellular migration, the cell monolayer was scratched using a blue pipette tip, followed by incubation with 30 ng/mL TNF-α for 24 h. Phase contrast images of the cells were acquired at 24 h. (**B**) The number of migrating cells was counted. The data are presented as the mean ± SEM of three independent experiments. (*** *p* < 0.001 vs. control cells at 0 h; ^#^ *p* < 0.05, ^##^ *p* < 0.01, compared to TNF-α-stimulated cells).

The role of COX-2 and MMP-9 during gastric inflammation and cell migration is well-established [36]. The primary objective of this study was to determine the effect of Lj-EtOH on TNF-α-induced functional alterations within GES-1 cells specifically mediated by COX-2 and MMP-9. A comprehensive analysis was conducted to determine the migratory behavior of GES-1 cells after a 24 h treatment with TNF-α. Lj-EtOH, a TNF-α antagonist, U0126, TSIIA, BAY 11-7082, MMP9i, or NS-398 pretreatment resulted in a significant inhibition of TNF-α-induced cell migration, as shown in Figure 5. Thus, Lj-EtOH plays a role in reducing cell migration by inhibiting the expression of COX-2 and MMP-9 expression in GES-1 cells.

## 4. Discussion

Lj belongs to the *Caprifoliaceae* family, also known as *Jin Yin Hua*, and is a commonly used traditional Chinese medicine, health supplements, cosmetics, and ornamental groundcover [1]. Lj could be used for functional/health supplement applications due to its intestinal benefits [37]. In addition to Lj, general natural products are also widely used in promoting human health through various ways, such as *Rhizoma polygonati* extract [38], Gentiana extract [39], camel milk [40], safranal [41], and so on. Numerous studies have identified over 140 component compounds, including essential oils, flavones, organic acids, iridoids, saponins, and inorganic elements. Lj and its constituents demonstrate diverse pharmacological effects including anti-inflammatory, antiviral, antibacterial (including against *Helicobacter pylori*), antioxidant, hepatoprotective, anticancer, immune-boosting functions, insecticidal, acaricidal, antipregnancy, antihyperlipidemic, antithrombotic, and anti-lipase activities [1,2,42]. In the present study, Lj-EtOH was examined based on its documented anti-inflammatory and gastroprotective properties. The induction of gastric inflammation has an important role in the development of gastric epithelial injury and the development of gastric inflammation-related disorders.

Chang et al. [43] found that LJ Flos (LJF) has been traditionally consumed orally as a medicinal plant and health food in China for years. To elucidate the gastrointestinal metabolism of LJF, three distinct in vitro models were used, specifically gastric juice, intestinal juice, and human intestinal bacteria. The identification of prototype compounds within the water extraction of LJF (LJF-WE) was accomplished through rigorous qualitative and quantitative analysis methodologies. They evaluated the stability of eight bioactive composites (sweroside, secoxyloganin, isochlorogenic acid B, neochlorogenic acid, chlorogenic acid, cryptochlorogenic acid, isochlorogenic acid A, and isochlorogenic acid C) in simulated gastric fluid, intestinal fluid, and human fecal bacteria. The results indicated that these compounds exhibited a higher degree of stability when subjected to gastric and intestinal fluids compared with the presence of fecal bacteria.

Bang et al. [31] examined the gastroprotective properties of BST-104, which is a water-based extract derived from LJ. They sought to elucidate the underlying mechanisms through the use of murine gastritis models induced by HCl/ethanol and gastric ulcer induced by acetic acid. The test subjects were orally administered BST-104, chlorogenic acid, or rebamipide, the latter serving as a positive control. The results indicated that BST-104 and its primary compound, chlorogenic acid, exhibited gastroprotective properties through their antioxidant activities, which involved enhanced levels of catalase, SOD, and GSH, whereas MDA levels were reduced. In addition, BST-104 and chlorogenic acid suppressed the secretion of proinflammatory cytokines (PGE_2_, TNF-α, IL-6, and IL-1β) by significantly downregulating the expression of NF-κB. Tang et al. [37] observed that Lj exhibited inhibitory effects on multiple cytokines, such as TNF-α, IL-1β, IL-6, IFN-γ, IL-12, and IL-17, in a murine model of DSS-induced ulcerative colitis. Furthermore, in a mouse model of induced immunosuppression through cyclophosphamide exposure, polysaccharide extracts derived from Lj successfully restored IL-2, TNF-α, and IFN-γ levels in the serum. This suggests that Lj’s polysaccharide extracts hold promise as immunomodulatory agents. Previous studies primarily concentrated on determining the regulatory impact of Lj on the well-being of animal intestines through the use of in vivo models, namely mice and rats, along with in vitro models, including HMC-1 cells and RAW 264.7 cells, whereas limited research has been conducted on other species and cell lines.

The precise mechanism by which Lj manifests its gastroprotective effectiveness against TNF-α-activated inflammation in gastric mucosal epithelial cells has yet to be elucidated. Gastric inflammation, which is caused by proinflammatory cytokines, such as TNF-α secreted by activated immune cells, is dependent on the activation of NF-κB and MMP-9 through NF-κB signaling [44]. Potter et al. [17] observed that a marked upregulation of COX-2 occurs in neoplastic sites or GC with inflammation. Moreover, *Helicobacter pylori* infection stimulates the TLRs/MyD88 and COX-2/PGE_2_ pathways, leading to the activation of NF-κB and subsequent induction of an inflammatory response in tumor tissues [18]. The mechanism of TNF-α-induced MMP-9 occurs via the MAPK pathway in different cell lineages in response to inflammatory mediators [45,46,47,48]. Furthermore, our study revealed that *H. zeylanica*-E_2_ inhibits the TNF-α-induced activation of the proinflammatory cPLA_2_/COX-2/PGE_2_ pathway in GC cells [19]. This suggests that the COX-2/PGE_2_ system may serve as an important pathological mediator in gastric inflammation-related disorders. In addition, we demonstrated that quercetin exhibits anti-inflammatory effects by suppressing the expression of TNF-α-stimulated MMP-9 in normal GES-1 cells [22]. In the study, we assessed the potential protective effect of Lj against damage induced by TNF-α on COX-2 and MMP-9 expression in normal GES-1 cells. The cells were stimulated by TNF-α, which caused a reduction in COX-2 and MMP-9 expression in GES-1 cells following treatment with Lj. The results indicate that Lj exhibits the capacity to uphold the structural soundness of the gastric mucosa and may represent a treatment for inflammation. Previous studies implicated the MAPK family, specifically MAPK (extracellular p38, JNK1/2, and ERK1/2), in the underlying pathogenic mechanisms of gastric inflammation and gastric ulcer [49]. Our results are consistent with these observations. In the present study, we explored the metastatic and inflammatory effects of Lj in normal GES-1 cells. Lj-EtOH ameliorated COX-2/PGE_2_ and MMP-9 damage activated by TNF-α in normal GES-1 cells, highlighting its potential role in immune modulation of TNF-α-mediated inflammation. To determine the interplay among TNFR, NF-κB, ERK1/2, c-Fos, COX-2, and MMP-9 upon the response provoked by TNF-α, we evaluated distinct inhibitors, which included a TNF-TNFR, NF-κB, ERK1/2, c-Fos, COX-2, and MMP-9 inhibitors.

The data from IF stain analysis revealed that stimulation of TNF-α resulted in the phosphorylation and translocation of NF-κB (p65) and the activities of NF-κB/p65-Luc and AP-1-Luc were mediated through pathways involving TNFR-ERK1/2-c-Fos and NF-κB. In addition, Lj caused a reduction in the expression of COX-2/PGE_2_ and MMP-9 induced by TNF-α in GES-1 cells and modulated the ERK1/2-c-Fos and NF-κB signaling. There is a plausible hypothesis suggesting that Lj effectively inhibits the activity of upstream molecules, such as TNFR, leading to the downstream inhibition of key signaling components, including NF-κB, ERK1/2, and c-Fos. The transcription factor NF-κB has a significant role in the modulation of inflammatory responses, given its involvement in the activation of proinflammatory cytokines. Following stimulation with these cytokines, NF-κB activity is activated, resulting from the phosphorylation and subsequent degradation of IκB. This results in the translocation of active NF-κB to the nucleus and stimulates the transcription of proinflammatory genes, cytokines, chemokines, adhesive proteins, and proteinases [50].

In the present study, we observed that pretreatment with BAY 11-7082 (NF-κB inhibitor) and TSIIA (c-Fos inhibitor) resulted in a reduction of TNF-α-induced COX-2/PGE_2_ and MMP-9 expression in GES-1 cells. COX-2/PGE_2_ and MMP-9 have emerged as promising targets for various therapies, which are the subject of many current studies [51,52]. Understanding the role of inflammatory mediators will be valuable for designing therapeutic strategies to treat gastric inflammatory diseases. There is the potential for mitigating gastric damage through the suppression of COX-2/PGE_2_ and MMP-9 production. Because of the widespread use of nonsteroidal anti-inflammatory drugs (NSAIDs), there is a need to discover substitute compounds to manage inflammation. Lj and its extracts may represent a promising alternative as dietary supplements for ameliorating the initial perturbations associated with gastric inflammation. Additionally, our findings indicate that TNF-α significantly induced an inflammatory response in GES-1 cells. On the other hand, IL-β or IFN-γ induced no significant effect. Therefore, we use TNF-α to focus on the role in gastrointestinal inflammation. Moreover, GES-1 belongs to the normal human gastric mucosa epithelial cell line, which is significantly close to normal physiological reactions. However, gastric cancer cell lines (such as AGS, AZ521, or TSGH) are far away from normal GES-1 cells to study gastric inflammatory-relative response.

From the data of the cell viability assay (Figure 2B,C), it seems that there existed a minimal effect on the viability of GES-1 cells upon treatment with Lj at concentrations ≤10 mM for 24 h. However, it is important to note that the safety and efficacy of Lj have not been definitively established, as indicated by previous studies [53]. The optimal dosage of Lj may differ based on an individual’s age, state of health, and additional factors. Although adverse effects resulting from Lj supplementation are infrequent, current data have not suggested an optimal dose range for Lj. The safety of natural products cannot always be assured, and determining a safe and effective dose is necessary [54,55]. However, an intravenous formulation containing Lj along with two other herbs has been safely administered to children for up to 7 days. When taken orally, Lj flower extracts are potentially safe for a duration of up to 8 weeks. Skin contact with Lj may cause allergic individuals to develop a rash [56]. Lj may exert anticoagulant properties and could interact with medications that also have anticoagulant effects, such as aspirin, plavix, cataflam, heparin, and coumadin, thus increasing the risk of bruising and bleeding. There is limited information regarding the use of Lj during pregnancy and breastfeeding, so it is advisable to avoid its use during these periods. Lj may have anti-inflammatory properties, although further studies are necessary to fully understand its mechanisms of action [57].

## 5. Conclusions

**Figure 6:**
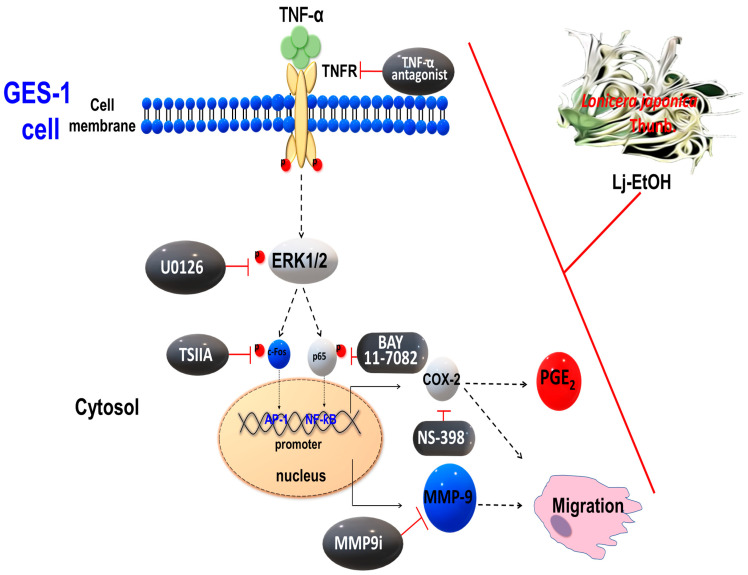
Diagram illustrating the effect of *Lonicera japonica* Thunb. ethanol extract on TNF-α-induced COX-2-derived PGE_2_ production and MMP-9 as well as the migration of normal GES-1 cells. Schematic diagram of the signaling pathways associated with the activity of *Lj*-EtOH extracts, which attenuated TNF-α-induced inflammation by downregulating COX-2, PGE_2_, and MMP-9 expression in normal GES-1 cells. *Lonicera japonica* Thunb. ethanol extract attenuates TNF-α-induced COX-2 and MMP-9 expressions in normal GES-1 cells through TNFR/ERK1/2/c-Fos and NF-κB pathways.

We examined the effect of Lj-EtOH on the metastatic and inflammatory properties of normal GES-1 cells in vitro. Lj-EtOH exhibits a protective effect against TNF-α-induced damage in normal GES-1 cells. Lj-EtOH specifically targeted the COX-2/PGE_2_ system and MMP-9 to ameliorate their detrimental effects. To elucidate the underlying mechanisms, we examined a series of inhibitors, including a TNF-α antagonist, U0126 (a selective inhibitor of ERK1/2), TSIIA (a specific inhibitor of c-Fos), and BAY 11-7082 (an inhibitor of NF-κB) and determined their effects on TNFR, ERK1/2, c-Fos, and NF-κB in response to TNF-α exposure. Lj-EtOH inhibits the TNF-α-induced expression of COX-2/PGE_2_ and MMP-9 in GES-1 cells. This effect is achieved through the inhibition of the proinflammatory TNFR-ERK1/2-c-Fos and NF-κB signaling pathways. Our results suggest that Lj-EtOH has potential as a therapeutic agent and warrants further consideration for normal gastric mucosal cells against disease-induced damage (see Figure 6. To identify the potential compounds in Lj-EtOH, the high-performance liquid chromatography profile and major compound structures were identified, as shown in Supplemental Figure S1. In addition, Lj-EtOH may be suitable as a food supplement to prevent early pathological emergence linked to gastric inflammation.

## Data Availability

The datasets generated for this study are available on request to the corresponding author.

## Supplementary Materials

The following supporting information can be downloaded at: https://www.mdpi.com/article/10.3390/cimb46070433/s1, Figure S1: The high-performance liquid chromatography profile and major compound structures.

## Abbreviations

AP-1: Activator protein-1; CCK-8: Cell Counting Kit-8; COX-2: Cyclooxygenase-2; ELISA: enzyme-linked immunosorbent assay; ERK: Extracellular-signal-regulated kinase; ERK2: Proto-oncogene extracellular-signal-regulated kinase 2; GAPDH: Glyceraldehyde-3-phosphate dehydrogenase; GES-1: Normal human gastric mucosa epithelial cell line; Lj: *Lonicera japonica* Thunb.; Lj-EtOH: *Lonicera japonica* Thunb. ethanol extract; Lj-H_2_O: *Lonicera japonica* Thunb. water extract; MMP-9: Matrix metallopeptidase-9; MMP9i: MMP-9 inhibitor; NF-κB: Nuclear factor kappa B; PGE_2_: prostaglandin E2; p-p65: Phospho-p65; p-ERK1/2: Phospho proto-oncogene extracellular-signal-regulated kinase 1/2; QRT-PCR: quantitative reverse transcription-polymerase chain reaction; TNF-α: Tumor necrosis factor-α; TNFR: Tumor necrosis factor-α receptor; TSIIA: Tanshinone IIA.
