# Supplementary material for: Lonicera japonica Thunb. Ethanol Extract Exerts a Protective Effect on Normal Human Gastric Epithelial Cells by Modulating the Activity of Tumor-Necrosis-Factor-α-Induced Inflammatory Cyclooxygenase 2/Prostaglandin E2 and Matrix Metalloproteinase 9
Source: Curr Issues Mol Biol. 2024 Jul 9;46(7):7303–23. doi: 10.3390/cimb46070433 (PMC11276375; doi:10.3390/cimb46070433)
Supplement: Supplementary file 1 [file cimb-46-00433-s001.zip › cimb-3064180-supplementary.pdf]

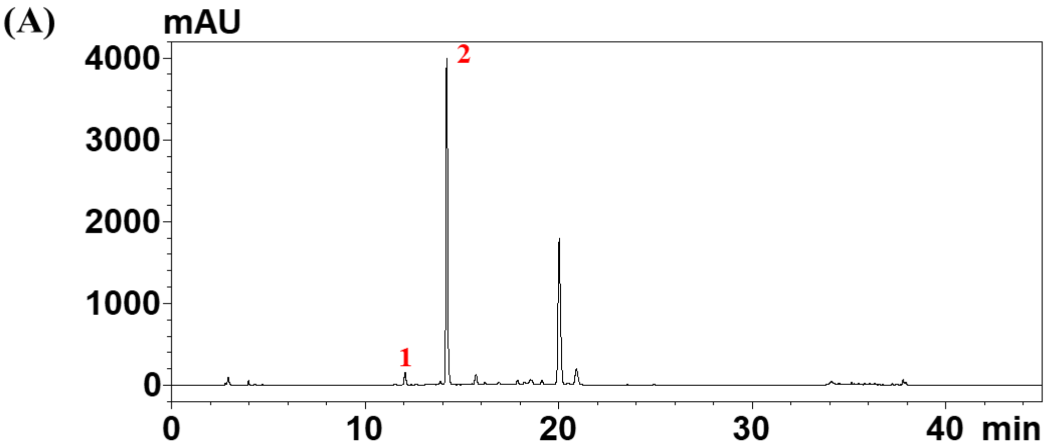

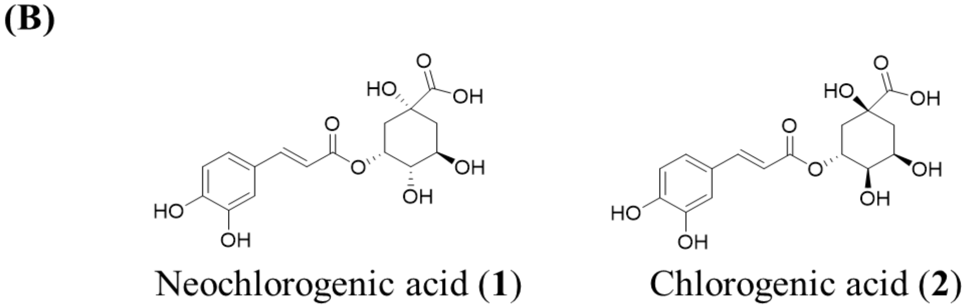

Supplemental Figure S1. The high-performance liquid chromatography profile and major compound structures. (A) High-performance liquid chromatography (HPLC) profile of the ethanolic crude extract of *L. japonica* Thunb. in 280 nm. (B) Chemical structure of major compounds. Neochlorogenic acid (1) and Chlorogenic acid (2).
